# Human coronavirus OC43 outbreak in wild chimpanzees, Côte d´Ivoire, 2016

**DOI:** 10.1038/s41426-018-0121-2

**Published:** 2018-06-27

**Authors:** Livia V. Patrono, Liran Samuni, Victor M. Corman, Leila Nourifar, Caroline Röthemeier, Roman M. Wittig, Christian Drosten, Sébastien Calvignac-Spencer, Fabian H. Leendertz

**Affiliations:** 1 Robert Koch-Institute, 13353 Berlin, Germany; 2 Max Planck Institute for Evolutionary Anthropology, 04103 Leipzig, Germany; 3 Taï Chimpanzee Project, Swiss Centre for Scientific Research, Abidjan, Côte d’Ivoire; 4 Charité - Universitätsmedizin Berlin, corporate member of Freie Universität Berlin, Humboldt-Universität zu Berlin, and Berlin Institute of Health, Institute of Virology, 10117 Berlin, Germany; 5 German Centre for Infection Research (DZIF), 10117 Berlin, Germany

Dear Editor,

A number of pathogens have been described to circulate between humans and non-human primates. The close relatedness between these hosts is thought to support pathogen transmission. Due to their rapid spread and difficult containment, airborne pathogens raise the greatest concerns. Common human respiratory viruses such as the human respiratory syncytial virus (HRSV), the human metapneumovirus (HMPV) and the human rhinovirus C, have caused lethal outbreaks in wild habituated great apes^1,2^. Strict prevention measures have been adopted to mitigate the risk of disease introduction at great ape research sites^3^, allowing habituation programmes to maximize positive effects on wildlife conservation^4^. However, transmission of infectious agents may still occur. Here we report the transmission of the human coronavirus (HCoV) OC43 to wild chimpanzees (*Pan troglodytes verus*) living in the Taï National Park, Côte d´Ivoire. These chimpanzees are habituated to human presence, and are in the focus of long-term observatory studies since the ’80s^5^. All members of the three communities (North, South and East) are individually known.

Between late December 2016 and early January 2017, a mild respiratory outbreak was observed in the East chimpanzee community (currently composed of 33 individuals). Daily monitoring by trained personnel identified sporadic coughing and sneezing throughout the group, mainly in morning hours. No other symptom was observed. Factors such as forest density, chimpanzee group fission–fusion and amount of time spent on the ground vs. on trees strongly influence whether individuals can be observed on a daily basis. In such conditions, the detection of disease onset and follow-up of symptoms’ course over time as well as the collection of samples is very complicated. During this outbreak, symptoms were reported once in at least nine individuals; of these, six were consistently identified as symptomatic on three consecutive days (Fig. 1, bold names). Fecal samples were collected as part of a continuous non-invasive health monitoring program^6^ and shipped to the Robert Koch Institute for analyses. To expand our window of investigation to non-outbreak times, a total of 59 samples collected from 18 individuals of this community between November 2016 and February 2017 were tested. This number reflects all available samples for the time frame of interest. We performed a PCR screening targeting major respiratory viruses including HRSV and HMPV, adenoviruses (AdVs), coronaviruses, enteroviruses, influenza A and B viruses, parainfluenza viruses and rhinoviruses^7,8^. PCR and sequencing identified the HCoV-OC43 in 14/59 samples, collected from 11 individuals, including those where symptoms were consistently reported (Fig. 1). HCoV-OC43 positive samples were collected within the time frame of observed respiratory disease outbreak, whereas samples collected before and after were negative. The detection in feces exclusively during the outbreak supports the hypothesis of this coronavirus being responsible for the observed mild respiratory symptoms. With the exception of AdVs, all other tests were negative. Adenoviruses were however detected in 49/59 samples across outbreak and non-outbreak times. Along with the evidence of AdVs being widely carried by wild great apes and shed in feces^9^, the continuous detection points towards an unlikely involvement in this outbreak.

**Fig. 1:**
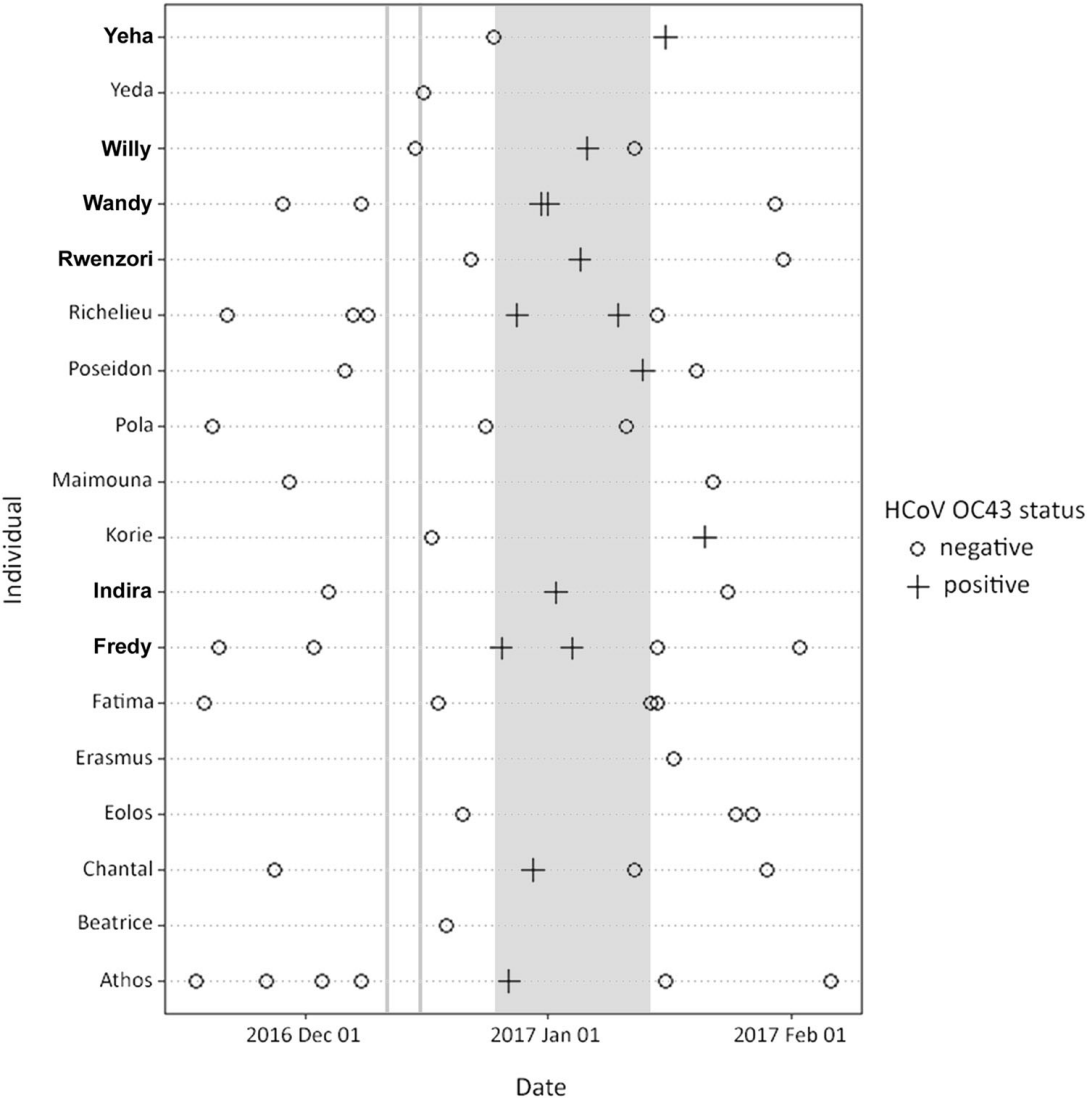
Human coronavirus OC43 detection in chimpanzee fecal samples over time. Each line represents an individual. Bold names represent consistently symptomatic individuals. Gray shadowing indicates the time frame of symptoms’ observation in the group. Vertical grey lines correspond to the dates in which human quarantine swabs were positive

Since 2008, the Taï Chimpanzee Project has implemented a mandatory quarantine for any individual intending to approach the chimpanzees^10^. To control for the potential onset of respiratory symptoms, all people entering the project area remain for 5 days in a camp separated from those used by research staff and are forbidden to enter the forest areas that correspond to chimpanzees’ territories. Additionally, on quarantine day 1 (QD1) and 5 (QD5), a throat swab is collected and stored in liquid nitrogen. Upon completion of the fifth day, individuals that did not develop symptoms can join the research camps and activities. According to current measures, swabs are tested for respiratory pathogens only in the event of an outbreak in the chimpanzees. To investigate whether HCoV-OC43 was circulating in humans at the time of the outbreak, 56 swabs collected between December 2016 and January 2017 were tested. Four swabs collected on QD1 and QD5 (10 and 14 December 2016, respectively) from two asymptomatic individuals were positive.

To strengthen epidemiologic links, the following two techniques were applied in parallel to obtain complete HCoV-OC43 genomes from feces: (i) a set of 24 nested PCR assays and sequencing of the amplicons and (ii) an in-solution hybridization capture approach followed by next-generation sequencing (supplementary methods). The two methods yielded comparable results, providing four complete (GenBank accession numbers MG977444, MG977445, MG977447 and MG977449) and three nearly complete viral genomes (GenBank accession numbers MG977446, MG977448 and MG977450). All consensus sequences were identical, suggesting that no mutations arose upon this episode of virus inter-species transmission and subsequent spread. Viral genome architecture corresponded to that previously reported for HCoV-OC43 viruses (supplementary figure 1). Hybridization capture applied on the HCoV-OC43 positive swabs collected during human quarantine (10 December 2016) identified two complete genomes: one differed from the genomes determined from chimpanzee fecal samples at 53 nucleotide positions (GenBank accession nr. MG977451) while the second was completely identical to these same genomes (GenBank accession nr. MG977452), thus providing the strongest possible genetic evidence of a local human-to-chimpanzee transmission. Phylogenetic analyses showed that the viruses identified in 2016 at Taï National Park fall within a clade of Asian HCoV-OC43 viruses isolated in 2012/2013 (supplementary figure 2). The lack of data on HCoVs circulating in Africa, and more generally the poor geographical sampling of HCoV-OC43 at any time, hampers a more accurate placement of these viruses within recent and contemporary strains.

To our knowledge, this is the first report of a HCoV causing a respiratory anthroponosis in wild great apes. The HCoV-OC43, which belongs to the species *Betacoronavirus 1* (BetaCoV1), is an endemic human pathogen and causes episodes of common cold in humans worldwide^11^. Beside in humans, BetaCoV1 strains were detected in highly divergent host species, including odd- and even-toed ungulates, carnivores and lagomorphs^12^. With the exception of SARS and MERS, human coronaviruses (HCoV-OC43, -NL63, -HKU1 and -229E) mainly cause mild infections in healthy individuals. It is therefore plausible that the virus was inadvertently introduced in the forest via asymptomatic shedding, overcoming the threshold of what quarantine *per se* can prevent. However, we cannot exclude other human sources of the infection in the forest, such as poachers.

Except for a recently identified human rhinovirus C in a wild chimpanzee^2^, anthroponotic transmissions to chimpanzees documented over the past decade were exclusively due to members of the virus family *Pneumoviridae*, resulting in high morbidities, overt clinical signs and frequent mortality. Disease severity may have played a role in raising awareness and attempting non-invasive monitoring of these specific pathogens. Given the mild clinical presentation reported here, it seems plausible that seasonal coronaviruses may have been transmitted to great apes on other occasions but were not diagnosed. Outbreak investigation is presumably only possible where daily observations and continuous sampling for health monitoring are implemented; overall exposure may nonetheless be retrospectively investigated using serology on non-invasive samples^13^.

Contrary to several previous examples of human respiratory viruses entering great ape populations, no mortality was recorded. In fatal HRSV and HMPV outbreaks, co-infections with opportunistic bacteria such as *Streptococcus pneumoniae* were detected and likely responsible for disease severity^14^. Despite the limited amount of the clinical data available for RNA viruses other than influenza, experimental evidence shows that infection with HRSV and HMPV favors bacterial colonization through multiple pathways^15^. The ability of triggering secondary infections has not been widely reported nor explored for seasonal coronaviruses, and could represent an essential factor influencing disease outcome.

The emergence of SARS and MERS has strikingly changed our perception of coronavirus pathogenicity and threat. There is no data supporting host-specific CoVs in great apes and other primates, but this report hints that anthroponotic transmission of HCoV-OC43 can result in respiratory disease in chimpanzees, revealing yet a new interface in coronavirus host switching. Surveillance programmes implemented for great ape conservation should thus systematically include coronaviruses. Ultimately, the establishment of on-site diagnostics to detect asymptomatic shedding could be a valuable asset to broaden the radius of action of quarantine.

## Electronic supplementary material

Supplementary figure 1

Supplementary figure 2

Patrono et al. Supplementary material
