# Supplementary material for: Human coronavirus OC43 outbreak in wild chimpanzees, Côte d´Ivoire, 2016
Source: Emerg Microbes Infect. 2018 Jun 27;7:118. doi: 10.1038/s41426-018-0121-2 (PMC6021434; doi:10.1038/s41426-018-0121-2)
Supplement: Supplementary file 1 — Supplementary figure 1 [file 41426_2018_121_MOESM1_ESM.pdf]

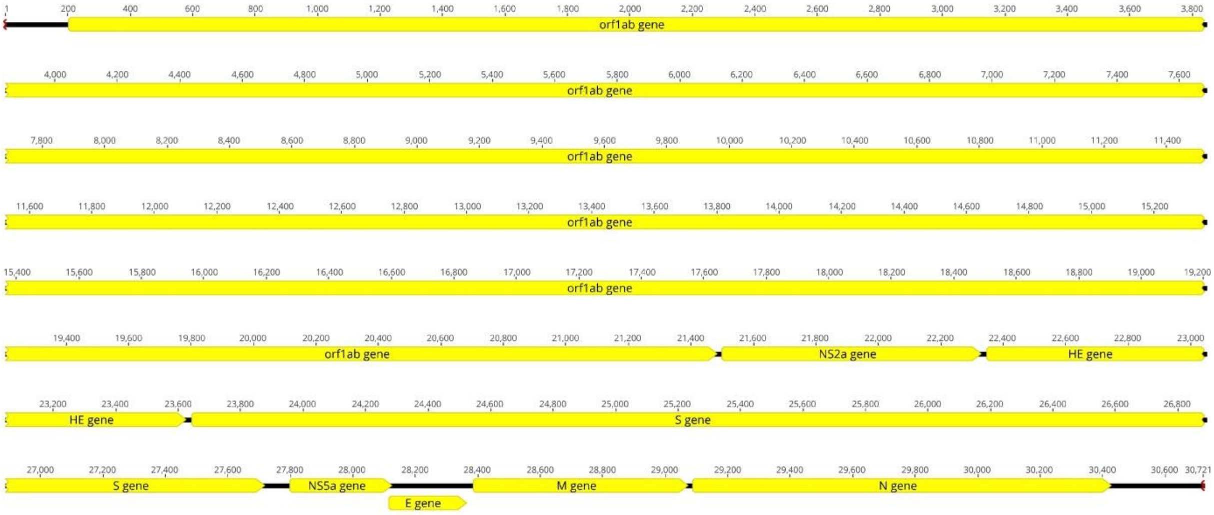

Supplementary figure 1: HCoV-OC43 genome architecture for prototype sequence MG977449. Yellow annotations represent the identified viral genes.
