# Supplementary material for: Human coronavirus OC43 outbreak in wild chimpanzees, Côte d´Ivoire, 2016
Source: Emerg Microbes Infect. 2018 Jun 27;7:118. doi: 10.1038/s41426-018-0121-2 (PMC6021434; doi:10.1038/s41426-018-0121-2)

Supplementary figure 2: Maximum likelihood tree of HCoV-OC43 genomes. Genomes identified in the 2016 outbreak at Taï National Park are in blue. Sequence MG977449 represents the identical virus detected in chimpanzee fecal samples and quarantine swabs of one human. Sequence MG977451 is the virus identified in the second human. Scale bar is expressed in substitution per variable sites. Inner branch colors represent branch support values (grey is ≤ 0.95; black is > 0.95). A strong correlation of root-to-tip distances and collection dates (R^2=0.80) revealed a strong temporal signal in this tree; the relatively long branches leading to sequences MG977449 and MG977451 can be explained by their evolution since the date of collection of their closest relatives in the tree (2012/2013).

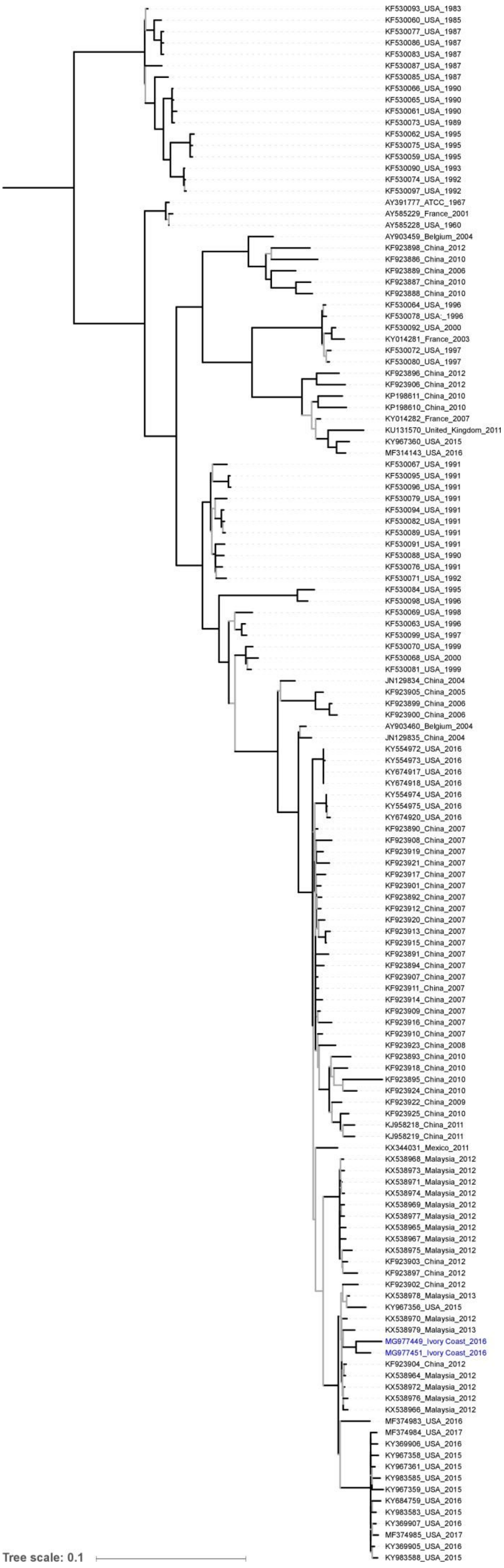

Supplement: Supplementary file 2 — Supplementary figure 2 [file 41426_2018_121_MOESM2_ESM.pdf]
