# Supplementary material for: Human coronavirus OC43 outbreak in wild chimpanzees, Côte d´Ivoire, 2016
Source: Emerg Microbes Infect. 2018 Jun 27;7:118. doi: 10.1038/s41426-018-0121-2 (PMC6021434; doi:10.1038/s41426-018-0121-2)
Supplement: Supplementary file 3 — Patrono et al. Supplementary material [file 41426_2018_121_MOESM3_ESM.docx]

**Supplementary information to the manuscript “Human coronavirus OC43 outbreak in wild chimpanzees, Côte d´Ivoire, 2016” by Patrono et al.**

Supplementary information provided as 3 separate files includes:

- Supplementary methods for HCoV-OC43 whole genome sequencing from fecal samples and phylogenetic analyses
- Two supplementary figures uploaded as individual files
- Supplementary figure legends

**Supplementary methods:**

**1. In-solution hybridization capture using MYbaits Custom Target Enrichment Kit performed at the Robert Koch Institute**

**1.1 Generating double stranded cDNA from RNA**

Total nucleic acids were extracted from approximately 60mg of feces using the GeneMATRIX Stool DNA Purification Kit (Roboklon). To reduce the DNA content, extracts were subject to DNase treatment using the TURBO DNA-free kit^TM^ (Ambion). Seventeen microliters (µl) of extract were mixed with 1 µl of DNase enzyme, 5µl of buffer and 27µl of nuclease free water and incubated at 37°C for 30 minutes (min). The enzyme was then inactivated by adding to the mix 5µl of DNase inactivation reagent. The RNA was purified using the MinElute PCR Purification kit (Qiagen), eluted in 10µl and transcribed into cDNA using the SuperScript IV reverse transcriptase (ThermoFisher). For this reaction, 8µl of RNA were mixed with 1µl of a 10µM random hexamer and 1µl of 10mM dNTPs, and incubated at 65°C for 5 min. Upon cooling on ice for 1 min, 4µl of 5X SSIV buffer, 1µl of DTT, 1µl of RNAaseOUT, 1µl of SSIV enzyme and 3µl of DPEC-treated water were added. The mix was then incubated according to the manufacturer’s instructions: 23°C for 10 min, 50°C for 10 min and 85°C for 10min. The resulting cDNA was double stranded by adding to the reaction 8µl of NEBNext Second Strand Synthesis Reaction Buffer, 4µl of Second Strand Synthesis Enzyme Mix and 48 Second Strand Synthesis Reaction Buffer of nuclease-free water (Second Strand Synthesis Module, New England Biolabs). Double stranded cDNA was purified using Ampure XP magnetic beads (Beckam-Coulter), eluted in 20µl low EDTA TE buffer and quantified using the Qubit High Sensitivity kit.

**1.2 Library preparation and in-solution capture**

Up to 10 ng of double stranded cDNA were used for each library. Each sample was fragmented using a Covaris S220 Focused-ultrasonicator® in a volume of 130µl low EDTA TE buffer using settings to generate a 400 bp fragment side (Intensity = 4, Duty cycle = 10%, Cycles per burst = 200, Treatment time = 55 s, Temperature = 7°C). Fragmented cDNA was then concentrated using the MinElute PCR purification kit and eluted into 50µl low EDTA TE buffer. Illumina compatible dual-index libraries were built from double stranded cDNA using the NEBNext Ultra II kit according to the standard protocol. No size selection was performed upon adapter ligation. Libraries were quantified using the KAPA HiFi library quantification kit and stored at -20°C until further use. For hybridization capture, libraries were pooled (150 ng of each) and concentrated using the MinElute PCR Purification kit (Qiagen) to a final volume of 10 µl. Two rounds of 24h hybridization capture at 65°C were performed using 2-fold tiling 80-mer RNA baits (Mybaits) designed to target seasonal respiratory coronaviruses (OC43, 229E, NL63 and HKU1). The myBaits Sequence Enrichment for Targeted Sequencing protocol (Version 2.3.1) was followed, with the exception that only a fourth of the recommended bait quantity was used per capture round. After each round of capture, the captured library pool was amplified using the KAPA Hot Start Library Amplification system to reach a minimum of 200ng of library and quantified using the KAPA HiFi Library Quantification Kit. The enriched pool was diluted to 4nM and sequenced on a MiSeq platform using the V3 chemistry (2x300 bp).

**1.3 Data analysis**

Low quality reads were removed from raw reads using Trimmomatic^1^. Trimmed reads were mapped to a HCoV-OC43 reference genome (accession number AY391777) using BWA-MEM^2^. We sorted mapping files and removed duplicates using the SortSam and MarkDuplicates tools from Picard (<http://broadinstitute.github.io/picard>). Consensus sequences were generated using Geneious v10.0.5^3^ by calling bases at positions covered by at least 2 reads and for which the majority of the reads agreed. Genomes were annotated in Geneious by applying existing annotations from other HCoV-OC43 genomes. All annotations were submitted to GenBank.

**2. Nested PCR System implemented at the Charité - Universitätsmedizin Berlin**

Complete or partial HCoV-OC43 genome sequences were generated from RNA, directly extracted from clinical specimens. Amplification and sequencing were done using sets of 24 nested PCR assays. These assays were designed to match and amplify about 1 to 1.2 KB overlapping fragments of HCoV-OC43 and other Betacoronavirus 1 sequences available in GenBank. Primer sequences are given in **Table 1**. The first round PCR used the SuperScript III OneStep RT-PCR kit (Life Technologies) in a 25-μL reaction volume, with 400 nmol of respective forward and reverse primers, 2x reaction buffer from the kit (containing 0.4 mM of each deoxyribonucleotide triphosphates and 3.2 mM magnesium sulfate), 0.4 μl of a 50 mM magnesium sulfate solution (Life Technologies), 1 μg of PCR-grade BSA (Roche), 1 μl of reverse transcriptase/Taq mixture, and 2 μL of RNA extract. The thermal cycling profile of the first round involved 20 min at 50°C for reverse transcription, followed by 3 min at 94°C, 45 cycles of 30 s at 95°C, 30 s at 56°C, and 90s at 68°C, with a final elongation step of 3 min at 68°C. A 25-μl second-round reaction was set up containing 1 μl of reaction mixture from the first round, 400 nmol of respective forward and reverse primers, 10x Platinum Taq Buffer (Life Technologies), 200 nmol of each deoxynucleotide triphosphates, 2.5 mmol of MgCl2, and 0.5U of Platinum Taq polymerase (Life Technologies). The amplification protocol of the second round involved 3 min at 94°C, 45 cycles of 30 s at 95°C, 30 s at 56°C, and 90s at 68°C, with a final elongation step of 3 min at 68°C. PCR products were visualized by agarose gel electrophoreses with ethidium bromide staining. All PCR products were either Sanger-sequenced (SEQLAB Sequence Laboratories, Göttingen, Germany) or sequenced on an Illumina MiSeq instrument using the Nextera XT DNA Library Preparation Kit and V3 chemistry (2x300 bp) according to manufacturer’s instructions.

**Table 1**

Oligonucleotide used for amplification and sequencing of HCoV-OC43 genome fragments:

| Primer ID | Sequence (5'-3') | Use |
| --- | --- | --- |
| BCoV1-Frag1-F | CCACTCCCTGTADTCTATGC | 1st round |
| BCoV1-Frag1-Fnest | TCAGCCAGGGACGTGTTG | 2nd round |
| BCoV1-Frag1-Rev | CCTACTGAAGAYTTGTGTRATG | 1st and 2nd round |
| BCoV1-Frag2-F | TATGTAGACCAGTATGGYTGTG | 1st round |
| BCoV1-Frag2-Fnest | TACTGGTRGTCTTGCAGWTGG | 2nd round |
| BCoV1-Frag2-Rev | CTTRGYTTTCTGAACTTGACTAG | 1st and 2nd round |
| BCoV1-Frag3-F | GGCCTTTCTKKTTTTAAGATTGGA | 1st round |
| BCoV1-Frag3-Fnest | TTAAGATTGGACGTARAAGAATTTGT | 2nd round |
| BCoV1-Frag3-Rev | CDCCATAAAAGAACATAGCATCC | 1st and 2nd round |
| BCoV1-Frag4-F | AATATWGTABTTYCACARGGTGG | 1st round |
| BCov1-Frag4-Fnest | TATGTKGCYGATTTTGCWTATTGG | 2nd round |
| BCoV1-Frag4-Rev | ACATTAACTCCATCACARAAYACATT | 1st and 2nd round |
| BCoV1-Frag5-F | GGTDBTTTTWATAAGGCYACTGTT | 1st round |
| BCoV1-Frag5-Fnest | YRTYTTGYTWACTGTTGATGGTGT | 2nd round |
| BCoV1-Frag5-Rev | AGGTCTATTAAAATATGTWARAGAA | 1st and 2nd round |
| BCoV1-Frag6-F | GATGTGGTGTTGGCTAMTGATG | 1st round |
| BCoV1-Frag6-Fnest | ACTTTTGGYAARCCTGTTATWTGG | 2nd round |
| BCoV1-Frag6-Rev | CATTTWACACGTAGACTRCGATTC | 1st and 2nd round |
| BCoV1-Frag7-F | TTATGCTTAGTACATTACATTGGAGT | 1st round |
| BCoV1-Frag7-Fnest | GTTACCAGCRCATGTKTTTATGAG | 2nd round |
| BCoV1-Frag7-Rev | TGCAACAMATGRTAACCATATC | 1st and 2nd round |
| BCoV1-Frag8-F | TWGTTGCTGTARTAGAYCAGGA | 1st round |
| BCoV1-Frag8-Fnest | GGCTCTACWGTGTTTAAYGTCC | 2nd round |
| BCoV1-Frag8-Rev | TTAACMACACCAAATGTATATTTTGG | 1st and 2nd round |
| BCoV1-Frag9-F | TGATCGTYTAAGYCTTACAGTGAT | 1st round |
| BCoV1-Frag9-Fnest | TTGTATGCTTRTKCTTACWGTGAC | 2nd round |
| BCoV1-Frag9-Rev | ATTGGVACACCTCCAATACCC | 1st and 2nd round |
| BCoV1-Frag10-F | TTGTTTAGAATGCCYTTGGGTG | 1st round |
| BCoV1-Frag10-Fnest | AGATATATGAATGCTAATGGATTGCG | 2nd round |
| BCoV1-Frag10-Rev | CATGGTCACACAACATTTTAACACA | 1st and 2nd round |
| BCoV1-Frag11-F | ACTGARTATGCTTCYAAYTCATC | 1st round |
| BCoV1-Frag11-Fnest | GTRGATCCTAAGAAAACGTATTTAG | 2nd round |
| BCoV1-Frag11-Rev | CACTAAAACARCAAGTGCGTAAATC | 1st and 2nd round |
| BCoV1-Frag12-F | AAGACTTGCTTTTATATGCTGCTGA | 1st round |
| BCoV1-Frag12-Fnest | CCAGCKTTGCATGTAGCTTC | 2nd round |
| BCoV1-Frag12-Rev | ACCYAGKTCATTGTACAACTTCT | 1st and 2nd round |
| BCoV1-Frag13-F | CTTGCMATAGATGCTTATCCACT | 1st round |
| BCoV1-Frag13-Fnest | CAYGAAAATGAAGAATACCAAAAGGT | 2nd round |
| BCoV1-Frag13-Rev | AGTWACAGTGTTAAARTATTTAGGTTC | 1st and 2nd round |
| BCoV1-Frag14-F | ATGCTCGTATTCGYGCTAAGC | 1st round |
| BCoV1-Frag14-Fnest | CTGCYCAATTGCCAGCACC | 2nd round |
| BCoV1-Frag14-Rev | CCAAACACCGTGTCATTATAGC | 1st and 2nd round |
| BCoV1-Frag15-F | CACTYATTGTTGATATTCAACAGTG | 1st round |
| BCoV1-Frag15-Fnest | TCRAGTAATCATGATWTRTATTGTAG | 2nd round |
| BCoV1-Frag15-Rev | AGAGCTAATAACACGRCTTTGTG | 1st and 2nd round |
| BCoV1-Frag16-F | CAGCTCTAACCAGAGCCCA | 1st round |
| BCoV1-Frag16-Fnest | ACAAGGTAATCTGGGGAGTAATG | 2nd round |
| BCoV1-Frag16-Rev | GARTAAACCATATCATTRATCTGG | 1st and 2nd round |
| BCoV1-Frag17-F | TGGAGAAATTCYACAGTTTGGAA | 1st round |
| BCoV1-Frag17-Fnest | TAGCCTBTTTGAYATGGCTAAATT | 2nd round |
| BCoV1-Frag17-Rev | GRCTGCGRTAHACAGCCAT | 1st and 2nd round |
| BCoV1-Frag18-F | AGGAGTTTTCAYTTTACHGATTTTTA | 1st round |
| BCoV1-Frag18-Fnest | ACKCCTWATYATGSCTTTAAATG | 2nd round |
| BCoV1-Frag18-Rev | CARCCTAGTAMCATTATCCWCCA | 1st and 2nd round |
| BCoV1-Hspike1-F | TACCAGTTATTTTGCTTGGCATTC | 1st round |
| BCoV1-Hspike1-Fnest | TTTATGGTGGATAATGTTACTAGG | 2nd round |
| BCoV1-Hspike1-Rev | AATCTCTTATTCCAAGTAGAAGGAT | 1st round |
| BCoV1-Hspike1-Rnest | CAAGTAGAAGGATYAAACCTGCT | 2nd round |
| BCoV1-Hspike2-F | GAATTGATACTACTGCAACAAGTTG | 1st round |
| BCov1-Hspike2-Fnest | CAGTTGTATTATAATTTACCTGCTG | 2nd round |
| BCov1-Hspike2-Rev | CTGATTTTCTGAGAGCAGTGG | 1st round |
| BCov1-Hspike2-Rnest | CAGTGGRGGCAACACTTTG | 2nd round |
| BCoV1-Hspike3-F | TAGGCAGCGAATGTAGTAAAGC | 1st round |
| BCoV1-Hspike3-Fnest | TAAAGTAAAGTTATCTGATGTCGGT | 2nd round |
| BCoV1-Hspike3-Rev | GTCGTCATGTGAAGTTTTGATTAC | 1st round |
| BCoV1-Hspike3-Rnest | GATTACTAACTCCTGGTATCCAG | 2nd round |
| BCoV1-Hspike4-F | GTTTTACTATTCTTCATATGCTGTTG | 1st round |
| BCoV1-Hspike4-Fnest | ACAGGATGTGGGACTAGTTGT | 2nd round |
| BCoV1-Hspike4-Rev | CCACATAAGCCACAAAATAATCATC | 1st round |
| BCoV1-Hspike4-Rnest | AACATAAACAAACATACTGCGAC | 2nd round |
| BCoV1-Frag19-F | AGTAGGATAYTGTAGAGTTCCT | 1st round |
| BCoV1-Frag19-Fnest | CTTGGTGYCGTAAYCAAGGT | 2nd round |
| BCoV1-Frag19-Rev | GTTCCMAGRTAGTAAAARTACCA | 1st and 2nd round |
| BCoV1-Frag20-F | GACAAGGTGTGCCTMTTGCA | 1st round |
| BCoV1-Frag20-Fnest | ACTGAAKCTAARGGRTAYTGGT | 2nd round |
| BCoV1-Frag20-Rev | CATTACHACACAAAAYTTTCAACTAAT | 1st and 2nd round |

**3. Phylogenetic analyses**

All available HCoV-OC43 complete genomes as of May 2018 (135) were downloaded from the National Center for Biotechnology Information (NCBI) database. Sequence MG977449 was included as a prototype sequence for all the viruses identified in the samples collected from the chimpanzees and one human. Sequence MG977451 from the second human was also included in the analyses. Sequences were aligned using MAFFT v7^4^ and collapsed to unique sequences using the online tool FaBox v1.4^5^. Identical sites and sites containing ambiguities (Ns) were stripped using Geneious v10.0.5, therefore only variable sites were included in the analysis. The best model of nucleotide evolution was identified using jModelTest v2.1.10^6^, applying the Bayesian information criterion (GTR+G). Phylogenetic analyses were performed in a maximum likelihood (ML) framework using PhyML v3^7^ as implemented in Seaview v3^8^, branch robustness was assessed using Shimodaira-Hasegawa approximate likelihood ratio tests^9^ (SH-like aLRT). To investigate the presence of a temporal signal, the resulting tree was analyzed using TempEst v1.5^10^ (http://tree.bio.ed.ac.uk/). The linear regression and R squared values were used to evaluate the correlation between tip dates and root-to-tip distances.

**Supplementary figure legends**

**1. Supplementary figure 1 legend**

HCoV-OC43 genome architecture for prototype sequence MG977449. Yellow annotations represent the identified viral genes.

**2. Supplementary figure 2 legend**

Maximum likelihood tree of HCoV-OC43 genomes. Genomes identified in the 2016 outbreak at Taï National Park are in blue. Sequence MG977449 represents the identical virus detected in chimpanzee fecal samples and quarantine swabs of one human. Sequence MG977451 is the virus identified in the second human. Scale bar is expressed in substitution per variable sites. Inner branch colors represent branch support values (grey is ≤ 0.95; black is > 0.95). A strong correlation of root-to-tip distances and collection dates (R^2^=0.80) revealed a strong temporal signal in this tree; the relatively long branches leading to sequences MG977449 and MG977451 can be explained by their evolution since the date of collection of their closest relatives in the tree (2012/2013).

References

1. Bolger, A. M., Lohse, M. & Usadel, B. Trimmomatic: A flexible trimmer for Illumina sequence data. *Bioinformatics* **30,** 2114–2120 (2014).

2. Li, H. & Durbin, R. Fast and accurate short read alignment with Burrows-Wheeler transform. *Bioinformatics* **25,** 1754–60 (2009).

3. Kearse, M. *et al.* Geneious Basic: An integrated and extendable desktop software platform for the organization and analysis of sequence data. *Bioinformatics* **28,** 1647–1649 (2012).

4. Katoh, K. & Standley, D. M. MAFFT multiple sequence alignment software version 7: Improvements in performance and usability. *Mol. Biol. Evol.* **30,** 772–780 (2013).

5. Villesen, P. FaBox: An online toolbox for FASTA sequences. *Mol. Ecol. Notes* **7,** 965–968 (2007).

6. Darriba, D., Taboada, G. L., Doallo, R. & Posada, D. Europe PMC Funders Group jModelTest 2 : more models , new heuristics and high- performance computing. *Nat. Methods* **9,** 6–9 (2015).

7. Guindon S, Dufayard JF, Lefort V, Anisimova M, Hordijk W, G. O. New algorithms and methods to estimate maximum-likelihood phylogenies: assessing the performance of PhyML 3.0. - PubMed - NCBI. *Syst. Biol.* **29,** 307–21 (2010).

8. Gouy, M., Guindon, S. & Gascuel, O. Sea view version 4: A multiplatform graphical user interface for sequence alignment and phylogenetic tree building. *Mol. Biol. Evol.* **27,** 221–224 (2010).

9. Anisimova, M., Gil, M., Dufayard, J. F., Dessimoz, C. & Gascuel, O. Survey of branch support methods demonstrates accuracy, power, and robustness of fast likelihood-based approximation schemes. *Syst. Biol.* **60,** 685–699 (2011).

10. Rambaut, A., Lam, T. T., Max Carvalho, L. & Pybus, O. G. Exploring the temporal structure of heterochronous sequences using TempEst (formerly Path-O-Gen). *Virus Evol.* **2,** vew007 (2016).
